# Labor values and consumption self-regulation among Chinese undergraduates: a mediation model of responsible and maladaptive consumption

**DOI:** 10.3389/fpsyg.2026.1961952

**Published:** 2026-10-22

**Authors:** Jingjie Pang, Yifan Guan, Ru Liu

**Affiliations:** 1 School of Marxism, Anhui Medical University, Hefei, China; 2 School of Marxism, Liaoning Normal University, Dalian, Liaoning, China; 3 School of Marxism, Qufu Normal University, Rizhao, Shandong, China

**Keywords:** college students, consumption self-regulation, financial wellbeing, impulsive consumption, labor values, sustainable consumption

## Abstract

**Introduction:**

This study examined the psychological mechanism through which institutional exposure to labor education is associated with college students' consumption orientations. Drawing on value internalization and consumer self-regulation perspectives, we tested whether labor values mediate the relationship between labor education exposure and responsible versus maladaptive consumption tendencies among Chinese undergraduates.

**Methods:**

A cross-sectional survey was conducted among 1,768 undergraduate students from 12 universities in China. Institutional exposure to labor education was assessed at the university level, while labor values and four consumption orientations—rational consumption, sustainable consumption, impulsive consumption, and credit-reliant consumption—were measured using structured questionnaires. Confirmatory factor analysis, regression analysis, cluster-robust standard errors, and bootstrap mediation analysis with 5,000 resamples were used.

**Results:**

Stronger institutional exposure to labor education was positively associated with students' labor values. Labor values were positively associated with rational and sustainable consumption and negatively associated with impulsive and credit-reliant consumption. Bootstrap analyses showed that labor values partially mediated the associations between institutional exposure to labor education and all four consumption outcomes. Exploratory heterogeneity analyses did not provide consistent evidence of significant differences across socioeconomic, regional, institutional, or disciplinary contexts.

**Discussion:**

The findings suggest that labor values may represent a psychological mechanism linking value-oriented educational exposure to consumption self-regulation. However, given the cross-sectional design and the limited number of universities, the findings should be interpreted as associational rather than causal.

## 1 Introduction

### 1.1 Psychological background: values, self-regulation, and consumption in emerging adulthood

Consumption behavior among college students is not only an economic issue but also a psychological and developmental one. Undergraduate students are typically situated in emerging adulthood, a transitional life stage characterized by identity exploration, increasing autonomy, and the gradual consolidation of long-term behavioral patterns (Arnett, 2000). During this period, students begin to make more independent consumption decisions, develop financial attitudes, and form habits that may influence their later financial wellbeing and psychosocial adjustment (Shim et al., 2009). Therefore, examining the psychological antecedents of college students' consumption orientations is important for understanding youth development, consumer wellbeing, financial wellbeing, and self-regulatory behavior (Consumer Financial Protection Bureau (CFPB), 2015; Mick et al., 2012).

In contemporary consumer environments, college students are exposed to multiple and sometimes conflicting consumption cues. On the one hand, rational and sustainable consumption reflects planning, delayed gratification, environmental concern, and consideration of long-term consequences. On the other hand, impulsive consumption and credit-reliant consumption are often associated with affective arousal, identity signaling, reduced self-control, and present-oriented decision-making (Belk, 1988; Dittmar et al., 1995; Rook, 1987; Baumeister et al., 1998; Vohs and Faber, 2007). The expansion of digital platforms, livestream commerce, and “buy now, pay later” services may further intensify the tension between responsible consumption and maladaptive consumption tendencies among young adults.

From a psychological perspective, values play an important role in shaping judgment and behavior. Values are relatively stable motivational beliefs that guide choices across situations and provide standards for evaluating actions (Schwartz, 1992). Prior research has shown that values are meaningfully associated with behavior, particularly when they are activated and central to the self-concept (Bardi and Schwartz, 2003; Verplanken and Holland, 2002). In this sense, labor values can be understood as work-related value orientations that emphasize the dignity of work, contribution, responsibility, fairness, and effort. These values may extend beyond the work domain and influence students' everyday decisions, including how they regulate consumption desires, evaluate material needs, and respond to short-term purchasing temptations.

### 1.2 Labor values as a psychological mechanism linking education and consumption

Labor values are closely related to the broader concept of work values in psychology and organizational behavior. Work values refer to beliefs about desirable goals, meanings, and outcomes associated with work and effort, and prior longitudinal evidence suggests that work values may have long-term implications for wellbeing (Dose, 1997; Fukasawa et al., 2020). They are often regarded as domain-specific expressions of broader personal values and may include intrinsic, extrinsic, social, and moral dimensions (Ros et al., 1999; Lyons et al., 2010). In the Chinese higher education context, labor values are commonly expressed through recognition of labor dignity, labor creation, labor contribution, and labor justice. Although these dimensions are culturally contextualized, they can be linked to internationally recognized psychological constructs such as work values, moral values, effort beliefs, and social responsibility.

Value-oriented education may contribute to the formation of such labor values. In higher education, labor education provides students with curricular learning, structured practice, social participation, and reflective evaluation related to work and responsibility. These educational experiences may function as institutionalized socialization inputs that shape students' value orientations. Consumer socialization research has long emphasized the role of family, peers, and media in shaping consumption-related attitudes and behavior (John, 1999). However, comparatively less attention has been paid to the role of higher education institutions as socialization agents that may influence consumption orientations through value formation.

The link between labor values and consumption behavior can be explained through consumer self-regulation. High self-control is associated with more adaptive outcomes across life domains, whereas reduced self-regulatory resources can increase impulsive buying tendencies (Tangney et al., 2004; Vohs and Faber, 2007). Labor values may function as a value-based self-regulatory resource by encouraging delayed gratification, effort-based reward beliefs, planned decision-making, and resistance to identity-driven or affectively triggered consumption. Students with stronger labor values may be more likely to evaluate consumption in relation to effort, necessity, social responsibility, and long-term wellbeing. As a result, they may show stronger rational and sustainable consumption orientations and weaker tendencies toward impulsive or credit-reliant consumption.

### 1.3 Research gaps and the present study

Although existing studies have provided important insights into values, consumer behavior, and youth financial wellbeing, several research gaps remain. First, consumer socialization research has mainly focused on family, peer, and media influences, while the role of institutionalized educational experiences in shaping consumption-related psychological mechanisms remains underexplored. Second, studies on college students' consumption often examine observable spending patterns or market behavior, but less attention has been paid to the psychological pathways through which value-oriented education may influence responsible and maladaptive consumption orientations. Third, although prior research has examined self-control, materialism, environmental values, and financial attitudes in relation to consumption behavior, relatively little is known about whether work-related values can serve as a psychological mechanism linking educational exposure to consumption self-regulation.

To address these gaps, the present study examines the relationship among institutional exposure to labor education, labor values, and college students' consumption orientations. Specifically, we conceptualize labor education as an institutionalized value-oriented educational input and labor values as a psychological mechanism of value internalization. Consumption orientations are examined in two directions: responsible consumption, including rational and sustainable consumption, and maladaptive consumption, including impulsive and credit-reliant consumption. By testing a mediation model among Chinese undergraduates, this study aims to clarify whether labor values mediate the association between educational exposure and consumption self-regulation.

This study contributes to the literature in three ways. First, it extends consumer socialization research by examining higher education institutions as potential agents of value-based consumer socialization. Second, it contributes to the psychology of consumption by identifying labor values as a potential self-regulatory mechanism associated with responsible and maladaptive consumption tendencies. Third, it provides empirical evidence from a large sample of Chinese undergraduates, thereby enriching the understanding of how culturally contextualized work-related values may relate to youth financial wellbeing and sustainable consumption.

### 1.4 Research questions

Based on the above theoretical framework, this study addresses the following research questions:

**RQ1:** Is institutional exposure to labor education positively associated with college students' labor values?

**RQ2:** Are labor values associated with stronger rational and sustainable consumption orientations and weaker impulsive and credit-reliant consumption tendencies?

**RQ3:** Do labor values mediate the relationship between institutional exposure to labor education and college students' consumption orientations?

**RQ4:** Are these associations heterogeneous across socioeconomic and institutional contexts?

## 2 Research questions and hypotheses

### 2.1 Theoretical framework

The present study is grounded in value internalization theory and consumer self-regulation perspectives. Values are motivational beliefs that guide individuals' judgments, choices, and behavioral tendencies across contexts, which is also consistent with broader perspectives emphasizing the role of beliefs and attitudes in shaping behavior (Ajzen, 1991). When values are internalized and become central to the self-concept, they are more likely to influence everyday decisions and behavioral regulation. In the context of this study, labor values are conceptualized as work-related value orientations that emphasize the dignity of labor, effort, contribution, fairness, and responsibility. These values may provide students with a psychological basis for evaluating consumption choices in relation to effort, necessity, long-term consequences, and social responsibility.

Institutional exposure to labor education may contribute to the development of labor values. Through curricular learning, structured practice, university–enterprise activities, and reflective evaluation, students may gradually internalize positive meanings attached to effort, work, and social contribution. From a socialization perspective, higher education institutions can function as value-forming environments that shape students' psychological orientations. Therefore, stronger and more systematic exposure to labor education may be associated with higher levels of labor values among college students.

Labor values may further influence consumption orientations through self-regulatory processes. Responsible consumption, including rational and sustainable consumption, requires planning, delayed gratification, consideration of long-term consequences, and concern for social or environmental outcomes. In contrast, maladaptive consumption, including impulsive and credit-reliant consumption, is often associated with immediate gratification, affective arousal, identity signaling, and weakened self-control. Students with stronger labor values may be more likely to connect consumption decisions with effort, responsibility, and future-oriented thinking, thereby showing stronger responsible consumption orientations and weaker maladaptive consumption tendencies.

Based on this logic, the present study proposes a mediation model in which institutional exposure to labor education is associated with students' labor values, which in turn predict responsible and maladaptive consumption orientations.

### 2.2 Research gaps

Although existing research has examined youth consumption, values, and financial wellbeing, several gaps remain.

First, previous studies on consumer socialization have mainly focused on family, peers, media, and digital platforms as sources of consumer attitudes and behavior. Less attention has been paid to higher education institutions as value-oriented socialization environments that may shape students' consumption-related psychological mechanisms.

Second, much of the existing research on college students' consumption emphasizes spending patterns, materialism, or financial literacy. Relatively few studies examine how value internalization may influence both responsible consumption and maladaptive consumption tendencies.

Third, although work values and labor-related values have been widely discussed in relation to career development, organizational behavior, and moral development, their potential influence on everyday consumption self-regulation remains insufficiently examined.

Fourth, there is limited empirical evidence on whether labor values mediate the relationship between educational exposure and consumption orientations among young adults. Addressing this gap may help explain how value-oriented education contributes not only to students' moral and work-related development but also to their consumer wellbeing and financial self-regulation. The proposed mediation model is illustrated in Figure 1.

**Figure 1:**
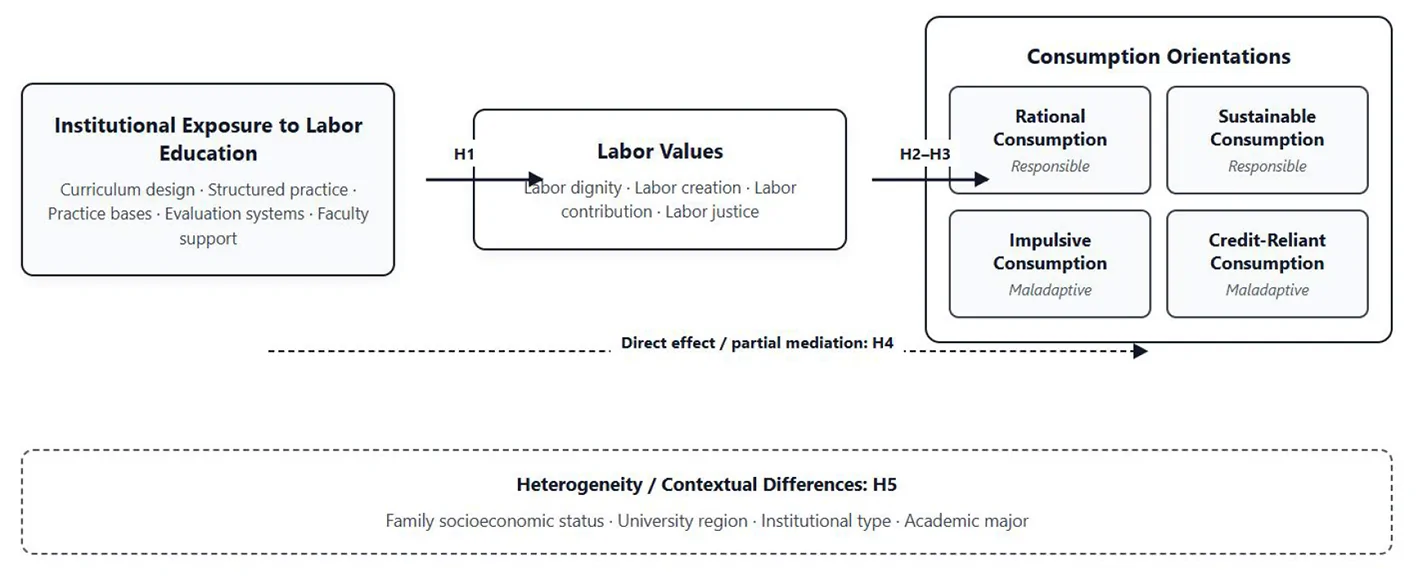
Proposed mediation model.

### 2.3 Key variables

#### 2.3.1 Institutional exposure to labor education

The independent variable is institutional exposure to labor education. In this study, it refers to the overall intensity and perceived quality of labor education that students receive in higher education institutions. It includes curriculum design, course hours or credits, structured practical activities, practice-base development, university–enterprise cooperation, evaluation systems, and faculty support.

Unlike general educational participation, institutional exposure to labor education is treated as a value-oriented educational input. Its purpose is not only to provide students with practical experience but also to cultivate positive meanings attached to labor, effort, responsibility, and social contribution.

#### 2.3.2 Labor values

The mediating variable is labor values. In line with the broader psychological concept of work values, labor values are defined as students' beliefs and evaluations concerning the meaning, dignity, contribution, and fairness of labor. In this study, labor values include four dimensions:

- Labor dignity: Recognition of the intrinsic value and dignity of labor;
- Labor creation: Belief that labor creates material and social value;
- Labor contribution: Endorsement of dedication, responsibility, and contribution to others or society;
- Labor justice: Concern for fairness in labor outcomes, opportunities, and social recognition.

These dimensions reflect a culturally contextualized form of work-related values and may serve as psychological resources for consumption self-regulation.

#### 2.3.3 Consumption orientations

The dependent variables are college students' consumption orientations. This study distinguishes between responsible and maladaptive consumption tendencies.

Responsible consumption includes:

- **Rational consumption:** Planned, need-based, and value-for-money-oriented consumption;
- **Sustainable consumption:** Environmentally and ethically oriented consumption that considers long-term and collective consequences.

Maladaptive consumption includes:

- **Impulsive consumption**: Unplanned and affectively triggered purchasing tendencies;
- **Credit-reliant consumption**: Anticipatory or debt-based consumption tendencies, such as spending beyond current financial capacity.

This distinction allows the study to examine whether labor values are associated with both the promotion of adaptive consumption tendencies and the inhibition of risky or maladaptive consumption tendencies.

### 2.4 Hypotheses

**H1: Institutional exposure to labor education and labor values**

Labor education provides students with structured opportunities to understand, experience, and reflect on the meaning of labor. Through repeated exposure to labor-related curricula, practical activities, and evaluation systems, students may internalize positive beliefs about effort, responsibility, contribution, and fairness. Therefore, stronger institutional exposure to labor education is expected to be associated with higher levels of labor values.

**H1:** Institutional exposure to labor education is positively associated with labor values among college students.

**H2: Labor values and responsible consumption**

Responsible consumption requires future-oriented thinking, self-control, and concern for broader social or environmental consequences. Students with stronger labor values may be more likely to recognize the relationship between effort, resources, and consumption. They may also be more inclined to evaluate purchases according to necessity, responsibility, and long-term wellbeing rather than immediate gratification. Therefore, labor values are expected to be positively associated with rational and sustainable consumption.

**H2a:** Labor values are positively associated with rational consumption among college students.

**H2b:** Labor values are positively associated with sustainable consumption among college students.

**H3: Labor values and maladaptive consumption**

Impulsive and credit-reliant consumption often reflects present-oriented decision-making, emotional stimulation, identity signaling, and weakened self-regulation. Labor values may help students resist such tendencies by strengthening effort-based reward beliefs, delayed gratification, and responsibility-oriented judgment. Students with stronger labor values may be less likely to engage in unplanned or credit-dependent consumption.

**H3a:** Labor values are negatively associated with impulsive consumption among college students.

**H3b:** Labor values are negatively associated with credit-reliant consumption among college students.

**H4: Mediating role of labor values**

Institutional exposure to labor education may influence consumption orientations indirectly through labor values. That is, labor education may first promote students' internalization of labor-related values, and these values may then guide their consumption self-regulation. Therefore, labor values are expected to mediate the relationship between institutional exposure to labor education and both responsible and maladaptive consumption tendencies.

**H4a:** Labor values mediate the relationship between institutional exposure to labor education and rational consumption.

**H4b:** Labor values mediate the relationship between institutional exposure to labor education and sustainable consumption.

**H4c:** Labor values mediate the relationship between institutional exposure to labor education and impulsive consumption.

**H4d:** Labor values mediate the relationship between institutional exposure to labor education and credit-reliant consumption.

**H5: Heterogeneity across socioeconomic and institutional contexts**

The associations among institutional exposure to labor education, labor values, and consumption orientations may differ across student and institutional backgrounds. Family socioeconomic status, university region, institutional type, and academic major may shape students' access to educational resources, value-socialization experiences, and consumption-related opportunities. Therefore, the strength of the proposed pathways may vary across socioeconomic and institutional contexts. However, given the limited prior evidence on these differences, the heterogeneity analyses were treated as exploratory.

**H5:** The relationships among institutional exposure to labor education, labor values, and consumption orientations may vary across socioeconomic and institutional contexts.

## 3 Method

### 3.1 Research design

This study adopted a cross-sectional survey design to examine the associations among institutional exposure to labor education, labor values, and college students' consumption orientations. The analytical framework followed an “educational exposure–value internalization–consumption self-regulation” logic. Institutional-level information on labor education was collected from university documents and administrative reports, whereas individual-level data were collected through structured questionnaires administered to undergraduate students.

Because students were nested within universities, the final dataset had a hierarchical structure. The main independent variable, institutional exposure to labor education, was constructed at the university level, whereas labor values and consumption orientations were measured at the student level. This design allowed us to examine whether institutional differences in labor education exposure were associated with students' value internalization and consumption-related psychological tendencies. Given the cross-sectional observational design, these associations should not be interpreted as evidence of causal effects or temporal ordering.

### 3.2 Participants and sampling procedure

The target population consisted of full-time undergraduate students enrolled in Chinese universities. A stratified cluster sampling strategy was used to improve sample heterogeneity across regions and institutional types. First, universities were stratified by regional economic development level, including eastern, central, and western China. Second, universities were further classified by institutional type, including “Double First-Class” universities and general undergraduate institutions. Within each stratum, universities were selected to ensure variation in educational resources and labor education implementation.

A total of 12 universities were included in the study. Within each university, undergraduate classes from different academic years and academic disciplines were selected as survey clusters. Questionnaires were distributed to 1,900 students. After excluding incomplete responses, patterned answers, and questionnaires with excessively short completion times, 1,768 valid responses were retained for analysis, yielding an effective response rate of 93.1%.

The final sample included 832 male students and 936 female students. Among them, 501 were first-year students, 486 were sophomores, 463 were juniors, and 318 were seniors or above. In terms of academic background, 618 students majored in humanities and social sciences, 762 in science, engineering, agriculture, or medicine, and 388 in economics, management, or arts. Regarding family socioeconomic status, 371 students reported low SES, 1,095 reported middle SES, and 302 reported high SES.

### 3.3 Ethical considerations

The requirement for ethical approval was waived by the Medical Ethics Review Committee of Chaohu Hospital Affiliated to Anhui Medical University because this study used an anonymous questionnaire survey, involved no intervention, collected no personally identifiable information, and posed minimal risk to participants. The study was conducted in accordance with the guidelines of the Medical Ethics Review Committee of Chaohu Hospital Affiliated to Anhui Medical University and the principles of the Declaration of Helsinki.

Participation was voluntary and anonymous. Before completing the questionnaire, all participants were informed of the purpose of the study, the approximate time required, the confidentiality of their responses, and their right to withdraw at any time without penalty. Informed consent was obtained from all participants before the survey. The collected data were used only for academic research purposes and were stored securely by the research team.

### 3.4 Measures

#### 3.4.1 Institutional exposure to labor education

Institutional exposure to labor education was measured using a Labor Education Implementation Intensity Assessment Form developed for this study. This measure was designed to capture the intensity and quality of labor education implementation at the university level. It consisted of five dimensions: curriculum design, structured practical instruction, practice-base development, evaluation system, and faculty support.

Curriculum design referred to labor education course hours, credits, and the degree to which labor education was embedded in undergraduate curricula. Structured practical instruction reflected the frequency, duration, and diversity of required labor-related practice activities. Practice-base development captured the number, stability, and utilization of university–enterprise or community-based practice bases. The evaluation system referred to whether labor education outcomes were formally assessed and how much weight they carried in student evaluation. Faculty support included the availability of specialized instructors, training frequency, and guidance quality.

Higher scores indicated stronger institutional exposure to labor education.

#### 3.4.2 Labor values

Labor values were measured as a multidimensional construct reflecting students' beliefs about the meaning, dignity, contribution, and fairness of labor. Based on prior research on work values and culturally contextualized labor education, the scale included four dimensions: labor dignity, labor creation, labor contribution, and labor justice.

Labor dignity refers to recognition of the intrinsic value and dignity of labor. Labor creation reflected the belief that labor produces material, social, and personal value. Labor contribution captured students' endorsement of responsibility, dedication, and contribution to others or society. Labor justice refers to concern for fairness in labor opportunities, outcomes, and social recognition.

Each dimension was measured using four items, resulting in 16 items in total. Responses were rated on a Likert-type scale, with higher scores indicating stronger labor values.

#### 3.4.3 Consumption orientations

Consumption orientations were measured using four subscales: rational consumption, sustainable consumption, impulsive consumption, and credit-reliant consumption.

Rational consumption reflected planned, need-based, and value-for-money-oriented purchasing tendencies. Sustainable consumption captured environmentally and ethically oriented consumption tendencies, including concern for long-term and collective consequences, which is consistent with research on pro-environmental and sustainable consumer behavior (Stern, 2000; Steg and Vlek, 2009; White et al., 2019). Impulsive consumption refers to unplanned, emotionally triggered, and temptation-driven purchasing tendencies. Credit-reliant consumption referred to anticipatory or debt-based consumption tendencies, including the inclination to spend beyond current financial capacity.

Each subscale contained four items. Higher scores indicated stronger tendencies toward the corresponding consumption orientation.

#### 3.4.4 Control variables

Several demographic and institutional variables were included as controls. At the individual level, we controlled for gender, year of study, academic major, and family socioeconomic status. At the institutional level, we controlled for university region and institutional type. These variables were included because prior research suggests that consumption orientations, financial attitudes, and value development may vary by demographic background and educational context.

### 3.5 Data collection procedure

Data were collected using a mixed online–offline approach over approximately 2 to 3 months. Institutional-level data were obtained from two sources. First, publicly available documents were collected from the websites of the Academic Affairs Office and Student Affairs Office of each sampled university, including labor education syllabi, credit requirements, curriculum documents, and practice-related policies. Second, a structured institutional information form was distributed to relevant administrative departments to collect additional information, such as the number of practice bases, university–enterprise cooperation programs, and annual funding for labor-related practical education.

Individual-level data were collected using an anonymous online questionnaire. Students in selected classes completed the questionnaire within a designated time window. To ensure data quality, the questionnaire included logic checks, attention checks, and completion-time screening. Responses with excessive missing values, patterned answering, or unrealistically short completion times were excluded. Individual-level data were then matched with institutional-level indicators according to the university attended by each respondent.

### 3.6 Scale scoring

Scale scores were calculated by averaging the corresponding item scores within each construct:


$$\begin{array}{l} X_{i}=\frac{1}{k}\overset{k}{\underset{m=1}{\sum }}x_{im} \end{array}$$

where *X*_*i*_ denotes the scale score for participant *i*, *x*_*im*_ denotes the score of item *m*, and *k* denotes the number of items in the corresponding scale.

The overall labor values score was calculated by averaging the four sub-dimension scores, including labor dignity, labor creation, labor contribution, and labor justice. Consumption orientation scores were calculated separately for rational consumption, sustainable consumption, impulsive consumption, and credit-reliant consumption.

### 3.7 Reliability and validity assessment

The reliability and validity of the core measurement instruments were assessed before hypothesis testing. Internal consistency reliability was evaluated using Cronbach's alpha (Cronbach, 1951):


$$\begin{array}{l} \alpha =\frac{k}{k-1}\left(1-\frac{\sum_{m=1}^{k}\sigma_{m}^{2}}{\sigma_{T}^{2}}\right) \end{array}$$

where *k* denotes the number of items, $\sigma_{m}^{2}$ denotes the variance of item *m*, and $\sigma_{T}^{2}$ denotes the variance of the total scale score.

Composite reliability was calculated using the standardized factor loadings:


$$\begin{array}{l} CR=\frac{{\left(\sum_{m=1}^{k}\lambda_{m}\right)}^{2}}{{\left(\sum_{m=1}^{k}\lambda_{m}\right)}^{2}+\sum_{m=1}^{k}\left(1-\lambda_{m}^{2}\right)} \end{array}$$

where λ_*m*_ denotes the standardized factor loading of item m.

Convergent validity was assessed using average variance extracted:


$$\begin{array}{l} AVE=\frac{\sum_{m=1}^{k}\lambda_{m}^{2}}{k} \end{array}$$

Discriminant validity was evaluated using the Fornell–Larcker criterion (Fornell and Larcker, 1981):


$$\begin{array}{l} \sqrt{AVE_{j}}>r_{jk} \end{array}$$

where $\sqrt{AVE_{j}}$ denotes the square root of AVE for construct *j*, and *r*_*jk*_ denotes the correlation between constructs j and k.

Confirmatory factor analysis was conducted to examine the measurement structure of labor values and consumption orientations. Model fit was evaluated using the chi-square/degrees of freedom ratio, comparative fit index, Tucker–Lewis index, root mean square error of approximation, and standardized root mean square residual.

### 3.8 Common method bias assessment

Because labor values and consumption orientations were measured using self-reported questionnaires, common method variance may have affected the associations among these individual-level constructs. However, institutional exposure to labor education was derived from university documents and administrative information rather than from the student self-report questionnaire, thereby reducing same-source bias for the association between institutional educational exposure and individual-level outcomes. Procedurally, the questionnaire was anonymous, item wording was kept clear and neutral, and participants were assured that there were no right or wrong answers. These procedures were intended to reduce social desirability concerns and evaluation apprehension.

Statistically, Harman's single-factor test was conducted as an initial diagnostic. In addition, a one-factor confirmatory factor analysis model was compared with the proposed multi-factor measurement model. If the one-factor model showed substantially poorer fit than the proposed measurement model, this would indicate that common method bias was unlikely to fully account for the observed relationships.

### 3.9 Data analysis

Data analysis was conducted in several steps. First, descriptive statistics and bivariate correlations were calculated for all core variables. Second, confirmatory factor analysis was conducted to evaluate the measurement model. Third, hierarchical regression analyses were used to test the associations among institutional exposure to labor education, labor values, and consumption orientations. Control variables were entered first, followed by the focal predictors.

The regression model predicting labor values was specified as follows:


$$\begin{array}{l} LV_{ij}=\beta_{0}+\beta_{1}ILE_{j}+\gamma {\text{X}}_{\text{i}\text{j}}+\delta {\text{Z}}_{\text{j}}+\varepsilon_{ij} \end{array}$$

where *LV*_*ij*_ denotes the labor values score of student *i* in university *j*, *ILE*_*j*_ denotes institutional exposure to labor education in university *j, Xij* denotes individual-level control variables, *Zj* denotes institutional-level control variables, and ε_*ij*_ den otes the error term.

The regression models predicting consumption orientations were specified as follows:


$$\begin{array}{l} Y_{kij}=\beta_{0}+\beta_{1}ILE_{j}+\beta_{2}LV_{ij}+\gamma {\text{X}}_{\text{i}\text{j}}+\delta {\text{Z}}_{\text{j}}+\varepsilon_{ij} \end{array}$$

where *Y*_*kij*_ denotes one of the four consumption outcomes:


$$\begin{array}{l} Y_{k}\in \left\{RC,SC,IC,CRC\right\} \end{array}$$

where RC denotes rational consumption, SC denotes sustainable consumption, IC denotes impulsive consumption, and CRC denotes credit-reliant consumption.

Mediation analyses were conducted using bias-corrected bootstrap procedures with 5,000 resamples (Hayes, 2022):


$$\begin{array}{l} M_{ij}=a_{0}+a_{1}X_{j}+\gamma {\text{C}}_{\text{i}\text{j}}+e_{ij}\\ Y_{ij}=b_{0}+c^{\prime }X_{j}+b_{1}M_{ij}+\gamma {\text{C}}_{\text{i}\text{j}}+u_{ij} \end{array}$$

The indirect effect was calculated as:


$$\begin{array}{l} \text{Indirect Effect}=a_{1}b_{1} \end{array}$$

The total effect was calculated as:


$$\begin{array}{l} \text{Total Effect}=c^{\prime }+a_{1}b_{1} \end{array}$$

The proportion mediated was calculated as:


$$\begin{array}{l} \text{Proportion Mediated}=\frac{a_{1}b_{1}}{c^{\prime }+a_{1}b_{1}} \end{array}$$

Because students were clustered within universities, standard errors were adjusted for clustering at the university level in the regression-based analyses. However, the institutional-level sample included only 12 universities. This limited number of higher-level units constrains the precision of institution-level inference and makes complex multilevel estimation less stable. Accordingly, coefficients involving institutional exposure to labor education were interpreted as cross-university associations rather than causal institutional effects, and the heterogeneity analyses were treated as exploratory. Exploratory heterogeneity analyses were conducted by family socioeconomic status, university region, institutional type, and academic major.

## 4 Results

### 4.1 Descriptive statistics

Descriptive statistics were first calculated to summarize the demographic characteristics of the sample. The final sample consisted of 1,768 undergraduate students from 12 universities in China. Among them, 832 were male students, accounting for 47.1%, and 936 were female students, accounting for 52.9%. In terms of year of study, 501 students were freshmen, 486 were sophomores, 463 were juniors, and 318 were seniors or above.

Regarding academic background, 618 students majored in humanities and social sciences, 762 majored in science, engineering, agriculture, or medicine, and 388 majored in economics, management, or arts. In terms of family socioeconomic status, 371 students reported low SES, 1,095 reported middle SES, and 302 reported high SES. The sample also showed regional and institutional diversity: 812 students were from universities in eastern China, 562 from central China, and 394 from western China. In addition, 707 students were enrolled in “Double First-Class” universities, whereas 1,061 were enrolled in general undergraduate institutions. The demographic characteristics of the sample are summarized in Table 1.

**Table 1:** Distribution of sample demographic characteristics (*N* = 1,768).

Variable	Category	Frequency	Percentage (%)
Gender	Male	832	47.1
	Female	936	52.9
Year of study	Freshman	501	28.3
	Sophomore	486	27.5
	Junior	463	26.2
	Senior and above	318	18.0
Major type	Humanities and social sciences	618	35.0
	Science, engineering, agriculture, and medicine	762	43.1
	Economics/ management and arts	388	21.9
Family SES(Subjective Perception)	Low	371	21.0
	Middle	1,095	61.9
	High	302	17.1
Region of the university	Eastern China	812	45.9
	Central China	562	31.8
	Western China	394	22.3
Type of University	“Double First-Class” university	707	40.0
	General undergraduate institution	1,061	60.0

### 4.2 Reliability and validity

The Cronbach's alpha coefficients ranged from 0.777 to 0.872, exceeding the commonly recommended threshold of 0.70. Specifically, the alpha coefficients for the four labor-value dimensions ranged from 0.811 to 0.872, whereas those for the four consumption-orientation dimensions ranged from 0.777 to 0.845. These results indicate acceptable internal consistency reliability. The reliability results for the core constructs are summarized in Table 2.

**Table 2:** Reliability test results for core variables.

Variable category	Latent construct	Number of items	Cronbach's α
Labor values	Labor dignity	4	0.843
Labor values	Labor creation	4	0.872
Labor values	Labor contribution	4	0.826
Labor values	Labor justice	4	0.811
Consumption behavior	Rational consumption	4	0.816
Consumption behavior	Sustainable consumption	4	0.845
Consumption behavior	Impulsive consumption	4	0.796
Consumption behavior	Credit-reliant consumption	4	0.777

Confirmatory factor analysis was conducted to evaluate the measurement structure of the core constructs. As shown in Table 3, standardized factor loadings ranged from 0.669 to 0.810. Composite reliability values ranged from 0.777 to 0.872. Most AVE values exceeded or approached the recommended 0.50 criterion. Although the AVE values for impulsive consumption and credit-reliant consumption were slightly below 0.50, their composite reliability values exceeded 0.70, and the standardized factor loadings were acceptable, suggesting adequate convergent validity overall.

**Table 3:** Convergent validity test results.

Latent construct	Item	Loading	CR	AVE
Labor dignity	LD1	0.718	0.843	0.574
	LD2	0.784		
	LD3	0.795		
	LD4	0.731		
Labor creation	LC1	0.780	0.872	0.630
	LC2	0.793		
	LC3	0.810		
	LC4	0.792		
Labor contribution	LCon1	0.742	0.827	0.544
	LCon2	0.738		
	LCon3	0.751		
	LCon4	0.718		
Labor justice	LJ1	0.727	0.811	0.518
	LJ2	0.738		
	LJ3	0.704		
	LJ4	0.710		
Rational consumption	RC1	0.757	0.817	0.527
	RC2	0.686		
	RC3	0.720		
	RC4	0.740		
Sustainable consumption	SC1	0.765	0.845	0.577
	SC2	0.746		
	SC3	0.770		
	SC4	0.757		
Impulsive consumption	IC1	0.702	0.797	0.495
	IC2	0.704		
	IC3	0.709		
	IC4	0.698		
Credit-reliant consumption	CRC1	0.680	0.777	0.465
	CRC2	0.697		
	CRC3	0.683		
	CRC4	0.669		

*p* < 0.001.

The proposed eight-factor measurement model showed acceptable model fit:


$$\begin{array}{l} \chi^{2}/df=1.075,\;CFI=0.999,\;TLI=0.998,\;RMSEA=\;0.007,\\ SRMR=0.016 \end{array}$$

Discriminant validity was assessed using the Fornell–Larcker criterion. As shown in Table 4, the square root of AVE for each construct was greater than its correlations with other constructs. The highest absolute inter-construct correlation was observed between labor dignity and labor contribution, *r* = 0.628, which remained lower than the square roots of AVE for the corresponding constructs. These results indicate satisfactory discriminant validity among the core constructs.

**Table 4:** Discriminant validity test results.

Construct	1	2	3	4	5	6	7	8
1. Labor dignity	**0.758**							
2. Labor creation	0.581	**0.794**						
3. Labor contribution	0.628	0.598	**0.737**					
4. Labor justice	0.532	0.455	0.540	**0.720**				
5. Rational consumption	0.357	0.433	0.406	0.349	**0.726**			
6. Sustainable consumption	0.460	0.429	0.458	0.394	0.549	**0.759**		
7. Impulsive consumption	−0.300	−0.328	−0.363	−0.290	−0.471	−0.446	**0.703**	
8. Credit-reliant consumption	−0.259	−0.331	−0.299	−0.279	−0.449	−0.406	0.615	**0.682**

Diagonal values (in bold) represent the square roots of AVE; off-diagonal values represent inter-construct correlation coefficients *p* < 0.001.

### 4.3 Common method bias

Because the individual-level variables were collected using self-reported questionnaires, common method bias was examined. Harman's single-factor test was first conducted as an initial diagnostic. The results showed that the first unrotated factor accounted for 25.928% of the total variance, which was below the commonly used 40% threshold.

In addition, a one-factor confirmatory factor analysis model was compared with the proposed eight-factor measurement model. The one-factor model showed substantially poorer fit:


$$\begin{array}{l} \chi^{2}/df=25.566,\;CFI=\;0.495,\;TLI=0.460,\;RMSEA=\;0.118,\\ \;SRMR=0.103 \end{array}$$

compared with the proposed eight-factor model:


$$\begin{array}{l} \chi^{2}/df=1.075,\;CFI=0.999,\;TLI=0.998,\;RMSEA=0.007,\\ \;SRMR=0.016 \end{array}$$

These results suggest that common method variance was unlikely to fully account for the observed associations. Nevertheless, these diagnostic tests cannot completely rule out common method or social-desirability bias associated with self-reported measures.

### 4.4 Correlations among core variables

As shown in Table 5, institutional exposure to labor education was positively correlated with labor values (*r* = 0.370, *p* < 0.001), rational consumption (*r* = 0.268, *p* < 0.001), and sustainable consumption (*r* = 0.283, *p* < 0.001), but negatively correlated with impulsive consumption (*r* = −0.246, *p* < 0.001) and credit-reliant consumption (*r* = −0.180, *p* < 0.001). Labor values were positively associated with rational consumption (*r* = 0.368, *p* < 0.001) and sustainable consumption (*r* = 0.425, *p* < 0.001), and negatively associated with impulsive consumption (*r* = −0.296, *p* < 0.001) and credit-reliant consumption (*r* = −0.264, *p* < 0.001).

**Table 5:** Means, standard deviations, and correlations among core variables.

Variable	M	SD	1	2	3	4	5	6
1. Institutional exposure to labor education	2.995	1.075	—					
2. Labor values	3.006	0.702	0.370^***^	—				
3. Rational consumption	3.006	0.912	0.268^***^	0.368^***^	—			
4. Sustainable consumption	2.988	0.955	0.283^***^	0.425^***^	0.394^***^	—		
5. Impulsive consumption	3.004	0.910	−0.246^***^	−0.296^***^	−0.318^***^	−0.311^***^	—	
6. Credit-reliant consumption	3.001	0.885	−0.180^***^	−0.264^***^	−0.298^***^	−0.275^***^	0.407^***^	—

^***^p < 0.001.

### 4.5 Institutional exposure to labor education and labor values

H1 predicted that institutional exposure to labor education would be positively associated with students' labor values. To test this hypothesis, hierarchical regression models were estimated with the overall labor values score and its four sub-dimensions as dependent variables. Gender, year of study, academic major, family socioeconomic status, university region, and institutional type were included as control variables. Standard errors were adjusted for clustering at the university level.

As shown in Table 6, institutional exposure to labor education was positively associated with the overall labor values score (β = 0.321, SE = 0.023, *p* < 0.001). It was also positively associated with all four dimensions of labor values: labor dignity (β = 0.249, SE = 0.030, *p* < 0.001), labor creation (β = 0.308, SE = 0.018, *p* < 0.001), labor contribution (β = 0.205, SE = 0.032, *p* < 0.001), and labor justice (β = 0.191, SE = 0.033, *p* < 0.001). These results indicate that students enrolled in universities with stronger labor education exposure reported higher levels of labor values. Therefore, H1 was supported.

**Table 6:** Regression results predicting labor values from institutional exposure to labor education.

Outcome	ILE β	SE	P	R^2^
Overall labor values	0.321	0.023	< 0.001	0.145
Labor dignity	0.249	0.030	< 0.001	0.079
Labor creation	0.308	0.018	< 0.001	0.110
Labor contribution	0.205	0.032	< 0.001	0.087
Labor justice	0.191	0.033	< 0.001	0.057

Standardized coefficients are reported. Gender, year of study, academic major, family SES, university region, and institutional type were included as control variables but are not shown for parsimony. Cluster-robust standard errors were used.

### 4.6 Labor values and consumption orientations

H2a and H2b predicted that labor values would be positively associated with responsible consumption orientations, including rational consumption and sustainable consumption. H3a and H3b predicted that labor values would be negatively associated with maladaptive consumption orientations, including impulsive consumption and credit-reliant consumption.

To test these hypotheses, four regression models were estimated with rational consumption, sustainable consumption, impulsive consumption, and credit-reliant consumption as dependent variables. Control variables and institutional exposure to labor education were included before entering labor values as the focal predictor.

As shown in Table 7, labor values were positively associated with rational consumption (β = 0.310, SE = 0.027, *p* < 0.001) and sustainable consumption (β = 0.373, SE = 0.024, *p* < 0.001). These findings suggest that students with stronger labor values were more likely to report planned, need-based, value-for-money-oriented consumption and environmentally or ethically oriented consumption. Thus, H2a and H2b were supported.

**Table 7:** Regression results predicting consumption orientations from labor values.

Outcome	Labor values β	SE	p	ILE β	SE	P
Rational consumption	0.310	0.027	< 0.001	0.140	0.031	< 0.001
Sustainable consumption	0.373	0.024	< 0.001	0.158	0.029	< 0.001
Impulsive consumption	−0.240	0.023	< 0.001	−0.184	0.026	< 0.001
Credit-reliant consumption	−0.232	0.020	< 0.001	−0.116	0.037	0.002

Standardized coefficients are reported. Gender, year of study, academic major, family SES, university region, institutional type, and institutional exposure to labor education were included as covariates. Control-variable coefficients are not shown for parsimony. Cluster-robust standard errors were used.

Labor values were negatively associated with impulsive consumption (β = −0.240, SE = 0.023, *p* < 0.001) and credit-reliant consumption (β = −0.232, SE = 0.020, *p* < 0.001). These findings indicate that students with stronger labor values were less likely to report unplanned, affect-driven purchasing tendencies and less likely to engage in credit-reliant consumption. Therefore, H3a and H3b were supported.

To further visualize the main regression pathways, the standardized mediation model is presented in Figure 2.

**Figure 2:**
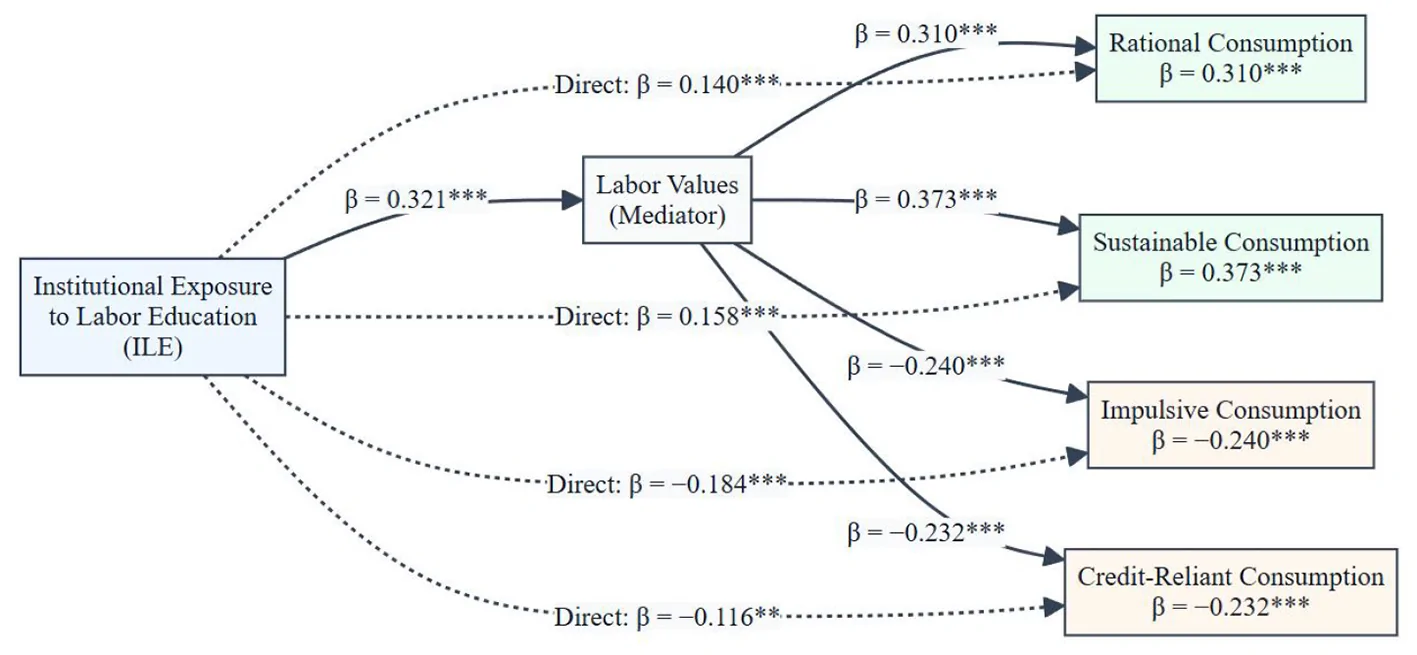
Standardized mediation model linking institutional exposure to labor education, labor values, and consumption orientations. ^**^p < 0.01; ^***^p < 0.001.

### 4.7 Mediation analyses

H4a–H4d predicted that labor values would mediate the relationships between institutional exposure to labor education and four consumption outcomes. Bootstrap mediation analyses were conducted with 5,000 resamples. Rational consumption, sustainable consumption, impulsive consumption, and credit-reliant consumption were specified as separate outcome variables. Gender, year of study, academic major, family socioeconomic status, university region, and institutional type were included as control variables.

As shown in Table 8, the indirect effects through labor values were significant for all four consumption outcomes. For rational consumption, the indirect effect was 0.100, with a 95% bootstrap confidence interval of [0.074, 0.129]. The direct effect remained significant (c′= 0.140), indicating partial mediation. The indirect effect accounted for 41.6% of the total effect. Thus, H4a was supported.

**Table 8:** Bootstrap mediation results for the indirect effects of labor values.

Outcome	Total effect	Direct effect	Indirect effect	Boot SE	95% CI for indirect effect	Proportion mediated
Rational consumption	0.239	0.140	0.100	0.014	[0.074, 0.129]	41.6%
Sustainable consumption	0.277	0.158	0.120	0.015	[0.092, 0.152]	43.2%
Impulsive consumption	−0.261	−0.184	−0.077	0.012	[−0.103, −0.056]	29.5%
Credit-reliant consumption	−0.190	−0.116	−0.075	0.011	[−0.099, −0.054]	39.2%

Standardized effects are reported. Bootstrap sample size = 5,000. Bias-corrected 95% confidence intervals are reported. Indirect effects are considered significant when the confidence interval does not include zero.

For sustainable consumption, the indirect effect was 0.120, with a 95% bootstrap confidence interval of [0.092, 0.152]. The direct effect remained significant (c′= 0.158), indicating partial mediation. The indirect effect accounted for 43.2% of the total effect. Thus, H4b was supported.

For impulsive consumption, the indirect effect was significant and negative (indirect effect = −0.077, 95% CI [−0.103, −0.056]). The direct effect remained significant (c′= −0.184), indicating partial mediation. The indirect effect accounted for 29.5% of the total effect. Thus, H4c was supported.

For credit-reliant consumption, the indirect effect was also significant and negative (indirect effect = −0.075, 95% CI [−0.099, −0.054]). The direct effect remained significant (c′= −0.116), indicating partial mediation. The indirect effect accounted for 39.2% of the total effect. Thus, H4d was supported.

The bootstrap indirect effects and their 95% confidence intervals are further visualized in Figure 3.

**Figure 3:**
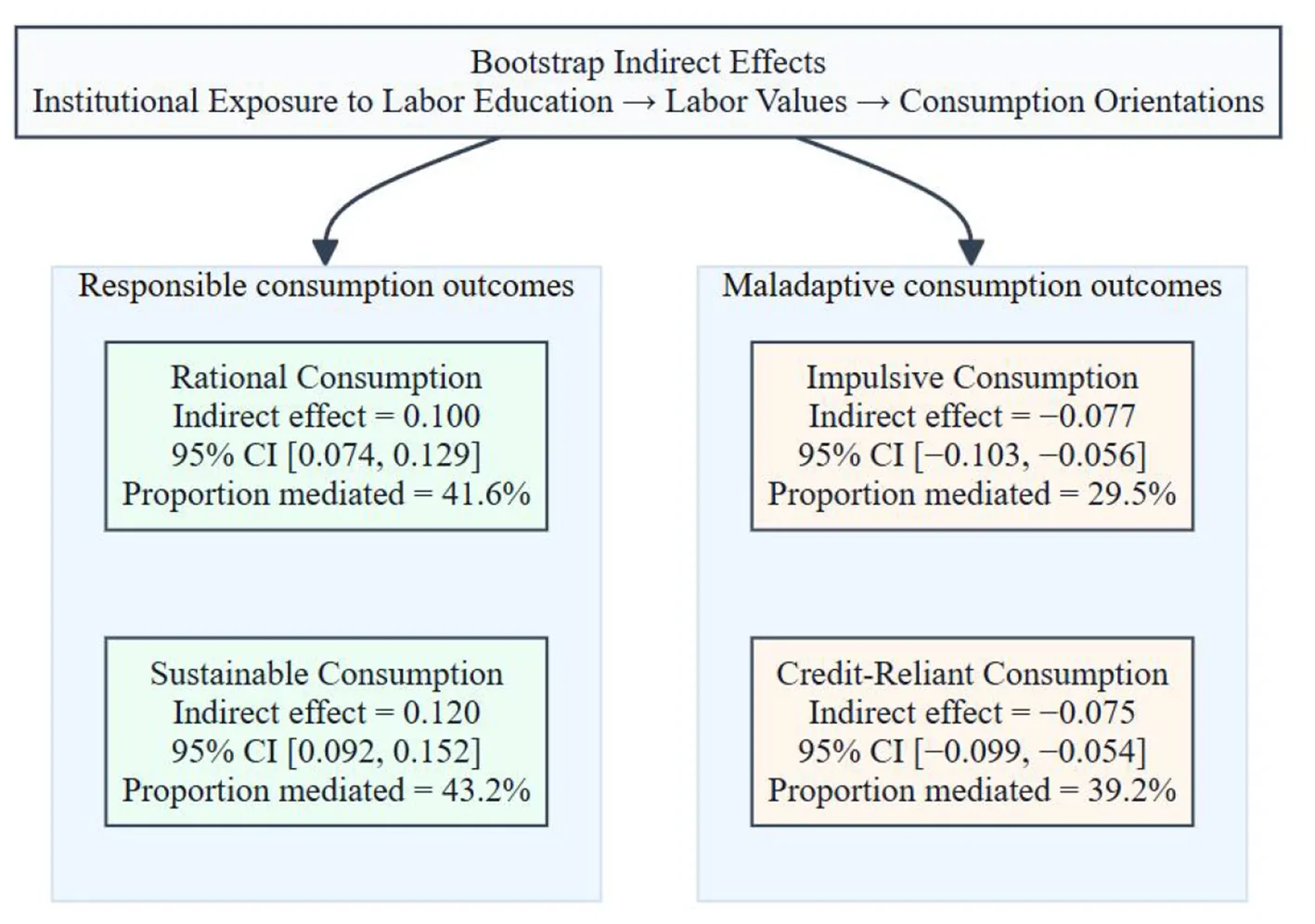
Bootstrap indirect effects through labor values.

### 4.8 Heterogeneity analyses

H5 predicted that the associations among institutional exposure to labor education, labor values, and consumption orientations would vary across socioeconomic and institutional contexts. To examine this possibility, interaction models were estimated by family socioeconomic status, university region, institutional type, and academic major.

As shown in Table 9, the interaction between institutional exposure to labor education and low family SES in predicting labor values was not statistically significant (β = −0.100, SE = 0.057, *p* = 0.082). Similarly, the interaction between institutional exposure to labor education and general undergraduate institution was not significant (β = −0.057, SE = 0.061, *p* = 0.352). The interactions between institutional exposure to labor education and the central/western region were also not significant in predicting rational consumption (β = −0.039, SE = 0.069, *p* = 0.574) or sustainable consumption (β = −0.032, SE = 0.038, *p* = 0.406). Finally, the interaction between labor values and STEM/agriculture/medicine majors in predicting sustainable consumption was not significant (β = 0.023, SE = 0.047, *p* = 0.621).

**Table 9:** Heterogeneity analyses of the main pathways.

Model	Outcome	Interaction term	β	SE	p	Interpretation
Model 1	Labor values	ILE × Low SES	−0.100	0.057	0.082	Not statistically significant
Model 2	Labor values	ILE × General undergraduate institution	−0.057	0.061	0.352	Not statistically significant
Model 3	Rational consumption	ILE × Central/Western region	−0.039	0.069	0.574	Not statistically significant
Model 4	Sustainable consumption	ILE × Central/Western region	−0.032	0.038	0.406	Not statistically significant
Model 5	Sustainable consumption	Labor values × STEM/agriculture/medicine	0.023	0.047	0.621	Not statistically significant

ILE, institutional exposure to labor education. Standardized regression coefficients are reported. Cluster-robust standard errors were used.

Taken together, the heterogeneity analyses did not provide consistent evidence that the proposed associations differed significantly across socioeconomic, regional, institutional, or disciplinary contexts. Therefore, H5 was not supported. Given the exploratory nature of these analyses and the limited number of participating universities, these null interaction effects should be interpreted cautiously and should not be taken as evidence of equivalence across groups.

## 5 Discussion

### 5.1 Theoretical contributions

This study examined whether labor values mediate the relationship between institutional exposure to labor education and college students' consumption orientations. The findings were consistent with the proposed “educational exposure–value internalization consumption self-regulation” framework, although the cross-sectional design does not establish the temporal or causal ordering of these relationships. Stronger institutional exposure to labor education was positively associated with students' labor values. Labor values were positively related to rational and sustainable consumption and negatively related to impulsive and credit-reliant consumption. The mediation analyses further showed that labor values partially mediated the associations between institutional exposure to labor education and all four consumption outcomes.

These findings extend consumer socialization research by highlighting higher education institutions as important value-forming environments. Previous research has often emphasized the role of family, peers, media, and digital platforms in shaping young people's consumption attitudes and behaviors. The present study suggests that institutionalized educational experiences may also be associated with students' consumption-related psychological orientations through the internalization of labor values. In this sense, higher education institutions may function not only as sites of knowledge transmission and skill development but also as environments in which students form value-based standards for everyday decision-making.

The findings also contribute to the psychology of consumption by identifying labor values as a potential self-regulatory resource. Responsible consumption requires planning, delayed gratification, and consideration of long-term consequences, whereas impulsive and credit-reliant consumption are often linked to immediate gratification, affective arousal, and weakened self-control. The observed associations suggest that students with stronger labor values may be more likely to evaluate consumption decisions in relation to effort, responsibility, necessity, and future wellbeing. Labor values may therefore function not only as work-related moral orientations but also as psychological resources relevant to everyday consumption self-regulation.

This study further broadens the theoretical scope of labor education by linking it to consumer wellbeing and youth financial self-regulation. Labor education is often discussed in relation to moral development, practical competence, and social responsibility. The present findings suggest that its potential influence may extend to students' consumption orientations, especially by promoting rational and sustainable consumption and reducing maladaptive consumption tendencies. This connection provides a basis for integrating value-oriented education with consumer behavior research and youth financial wellbeing studies.

The heterogeneity analyses did not detect statistically significant differences across the socioeconomic, regional, institutional, or disciplinary groups examined. However, these null interaction effects should not be interpreted as evidence that the associations are equivalent across groups. Given the limited number of participating universities and the exploratory nature of these analyses, statistical power to detect contextual differences may have been limited. Further studies with larger and more diverse institutional samples are needed to evaluate the generalizability and potential heterogeneity of these associations.

### 5.2 Practical implications

The heterogeneity analyses did not detect statistically significant differences across the socioeconomic, regional, institutional, or disciplinary groups examined. However, these null interaction effects should not be interpreted as evidence that the associations are equivalent across groups. Given the limited number of participating universities and the exploratory nature of these analyses, statistical power to detect contextual differences may have been limited. Further studies with larger and more diverse institutional samples are needed to evaluate the generalizability and potential heterogeneity of these associations. The positive association between institutional exposure to labor education and labor values suggests that labor education should not be limited to fragmented activities or symbolic participation. A more coherent institutional arrangement may help students better understand the meaning of labor, responsibility, contribution, and fairness.

Labor education should place greater emphasis on value internalization rather than mere task completion. Practical activities are educationally meaningful only when students are guided to reflect on the relationship between labor, effort, resources, and social responsibility. Universities may combine labor-related practice with reflective writing, group discussion, project-based learning, and formative assessment. These approaches may help students transform external educational exposure into internalized labor values that are relevant to everyday judgment and behavior.

The findings also suggest that labor education can be connected with consumer education and financial literacy education. Because labor values were associated with stronger rational and sustainable consumption and weaker impulsive and credit-reliant consumption, labor education may provide a value-based foundation for helping students develop healthier consumption habits. Educational modules may connect labor experience with topics such as budgeting, responsible spending, delayed gratification, sustainable consumption, and the risks of excessive credit use. Such integration would allow labor education to contribute not only to moral and practical development but also to students' financial wellbeing.

Although the heterogeneity analyses did not show significant subgroup differences, universities should still ensure that high-quality labor education resources are accessible to all students. Although no statistically significant moderation effects were detected, these results should be interpreted cautiously because the institutional sample was limited to 12 universities. The present findings therefore do not establish that the observed associations are equivalent across socioeconomic or institutional contexts. Nevertheless, ensuring broad access to high-quality labor education remains a reasonable educational consideration. Policy efforts should therefore focus on improving the overall quality, continuity, and inclusiveness of labor education rather than limiting attention to particular student groups.

### 5.3 Limitations and future directions

Several limitations should be acknowledged. The cross-sectional design limits causal inference. Although the proposed mediation model was theoretically grounded and statistically supported, the temporal order among institutional exposure to labor education, labor values, and consumption orientations cannot be fully established. Accordingly, the statistical mediation findings should be interpreted as evidence of indirect associations consistent with the proposed theoretical model rather than as confirmation of a causal mediation process. Future studies could adopt longitudinal designs, quasi-experimental approaches, or intervention studies to examine whether changes in labor education exposure lead to subsequent changes in labor values and consumption behavior.

The core individual-level variables were measured using self-reported questionnaires. Although reliability, validity, and common method bias tests indicated acceptable measurement quality, self-report data may still be influenced by social desirability, recall bias, or subjective interpretation. Although the institutional exposure measure was obtained independently from university-level documentary and administrative sources, the associations between labor values and consumption orientations may still be affected by common method variance because both were self-reported by students. Future research could incorporate multi-source data, such as administrative records of labor education participation, teacher evaluations, behavioral consumption tasks, or actual spending records, to provide more objective evidence.

A further limitation concerns the institutional sample. Although the study included a relatively large number of individual participants, these students were drawn from only 12 universities. Because institutional exposure to labor education varied at the university level, the effective number of institutional units was therefore limited. This restricts the precision of institution-level inference, reduces statistical power for detecting cross-institutional heterogeneity, and limits the extent to which the participating universities can be considered representative of Chinese higher education as a whole. Future studies should include a larger and more diverse sample of universities and, where sufficient institutional units are available, employ multilevel designs to more clearly distinguish student-level and institution-level variation.

In addition to the limited institutional sample, the study focused exclusively on Chinese undergraduate students, which may restrict generalizability to other educational stages and cultural contexts. Specific educational, cultural, and institutional environments shape labor values. Future research could examine whether similar mechanisms operate among vocational students, graduate students, or students in other national contexts. Cross-cultural comparison would be particularly useful for clarifying which aspects of labor values are culturally specific and which are more broadly applicable.

Although this study examined several consumption orientations, it did not directly measure actual consumption behavior. Rational consumption, sustainable consumption, impulsive consumption, and credit-reliant consumption were assessed as psychological tendencies. Future studies could combine questionnaire measures with behavioral indicators, such as spending logs, online purchase records, experimental impulse-buying tasks, or credit-use behavior, to better evaluate the behavioral consequences of labor values.

The exploratory heterogeneity analyses did not detect statistically significant differences across the examined socioeconomic, regional, institutional, or disciplinary contexts. Given the limited number of universities and the resulting statistical power for institution-level comparisons, these null findings should not be interpreted as evidence of equivalence across groups. Future research may further examine whether other contextual factors moderate the proposed pathway, such as digital consumption exposure, financial literacy, peer consumption norms, materialism, family financial socialization, or platform-based credit use. These variables may help explain when and for whom labor values are more strongly associated with consumption self-regulation.

## 6 Conclusion

This study examined the relationship among institutional exposure to labor education, labor values, and college students' consumption orientations. The findings showed that stronger institutional exposure to labor education was positively associated with students' labor values. Labor values, in turn, were positively associated with rational and sustainable consumption and negatively associated with impulsive and credit-reliant consumption. These results are consistent with the proposed “institutional exposure–value internalization–consumption self-regulation” framework.

The mediation analyses further indicated that labor values partially mediated the associations between institutional exposure to labor education and all four consumption outcomes. This suggests that labor education may be related to students' consumption orientations not only through direct institutional influence but also through the internalization of labor-related values. By emphasizing effort, responsibility, contribution, and fairness, labor values may help students form more planned, sustainable, and self-regulated consumption orientations.

The heterogeneity analyses did not detect statistically significant differences across the examined socioeconomic, regional, institutional, or disciplinary contexts. However, given the limited institutional sample, these findings should not be interpreted as demonstrating that the proposed associations are invariant across groups.

Overall, this study highlights the relevance of labor education for consumer wellbeing and youth financial self-regulation. The findings suggest that systematic labor education may be associated with value orientations that are relevant to responsible consumption and lower maladaptive consumption tendencies. Future research using longitudinal, quasi-experimental, or behavioral data is needed to further clarify causal relationships and examine how labor values influence actual consumption behavior over time.

## Data Availability

The raw data supporting the conclusions of this article will be made available by the authors, without undue reservation.
